# Comparison of Three Methods for Wind Turbine Capacity Factor Estimation

**DOI:** 10.1155/2014/805238

**Published:** 2014-01-22

**Authors:** Y. Ditkovich, A. Kuperman

**Affiliations:** Hybrid Energy Sources Laboratory, Department of Electrical Engineering and Electronics, Ariel University Center of Samaria, 40700 Ariel, Israel

## Abstract

Three approaches to calculating capacity factor of fixed speed wind turbines are reviewed and compared using a case study. The first “quasiexact” approach utilizes discrete wind raw data (in the histogram form) and manufacturer-provided turbine power curve (also in discrete form) to numerically calculate the capacity factor. On the other hand, the second “analytic” approach employs a continuous probability distribution function, fitted to the wind data as well as continuous turbine power curve, resulting from double polynomial fitting of manufacturer-provided power curve data. The latter approach, while being an approximation, can be solved analytically thus providing a valuable insight into aspects, affecting the capacity factor. Moreover, several other merits of wind turbine performance may be derived based on the analytical approach. The third “approximate” approach, valid in case of Rayleigh winds only, employs a nonlinear approximation of the capacity factor versus average wind speed curve, only requiring rated power and rotor diameter of the turbine. It is shown that the results obtained by employing the three approaches are very close, enforcing the validity of the analytically derived approximations, which may be used for wind turbine performance evaluation.

## 1. Introduction

Since the wind blows inconsistently, wind turbines barely operate at their rated power. Therefore the concept of capacity factor (CF) is usually engaged to assess the expected wind turbine energy delivery [1]. In order to calculate the CF, wind probability distribution function (PDF) and turbine power performance curve are required. The capacity factor, given by the ration between the average and rated turbine power, is usually formulated as

(1) $$\begin{array}{c} \text{CF}=\frac{E\left[P\right]}{P_{R}}=\frac{1}{P_{R}}\overset{\infty }{\underset{0}{\int }}P\left(v\right)f\left(v\right)dv, \end{array}$$

where *E*[·] is the mean value operator, *P*(*v*) and *P*_*R*_ are the power curve and rated power of the turbine, respectively, and *f*(*v*) is the wind PDF. In reality, the power curve of the turbine is a discrete series rather than a continuous function, and the wind PDF in (1) is the result of fitting the discrete raw wind data to an *a priori* assumed PDF. Hence, (1) is actually an approximation. Nevertheless, several *analytic* solutions of (1) have been presented in the literature [2, 3], allowing thorough understanding of the factors, affecting the CF. The more accurate (referred to as *quasiexact* thereafter) solution, taking into account the original rather than processed wind speed and power curve data, exists in a spreadsheet form only [4] and its outcome is given by a numerical value without any insight into the CF formation. Nevertheless, both mentioned methods are not much of a help when very little is known about a site and wind turbine and it is desired to quickly and fairly accurately predict the annual energy yield. A pretty handy and accurate (within 10%) solution was proposed in [4], where the authors demonstrated that, for midrange Rayleigh winds (5–9 m/s), the CF is a linear function of the average wind speed, depending on the turbine rated power and rotor blade diameter only. Unfortunately, the linear approximation is no longer valid for the winds outside the mentioned range and a nonlinear approximation of CF valid in the whole feasible speed range was proposed in [5].

In this paper, the three approaches to calculating the CF are reviewed and then compared using a case study. It is shown that the obtained results are similar, justifying the use of analytically derived relations in spite of the fact that they describe an approximate solution only. Hence, analytical derivations of additional merits of wind turbine performances which rely on capacity factor (such as Turbine Performance Index [6, 7] and Turbine-Site Pairing Performance [8]) are justified and the results obtained using these relations are reliable.

## 2. The Wind Data 

**Figure 1:**
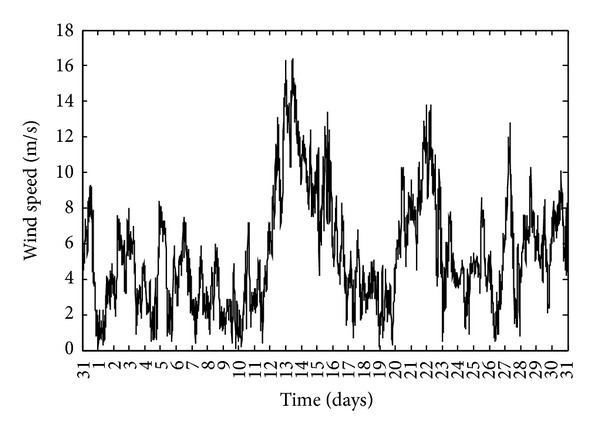
A typical monthly wind speed raw data.

The wind speed data is usually provided by meteorological stations as raw matrix of wind speed versus time at 10 m height, while sample times vary from 10 minutes to 1 day. In reality, the sample time is much higher than stated and the available data sample is actually an average of tens to thousands of faster samples. An example of monthly wind speed raw data represented by 10-minute samples is shown in Figure 1.

**Figure 2:**
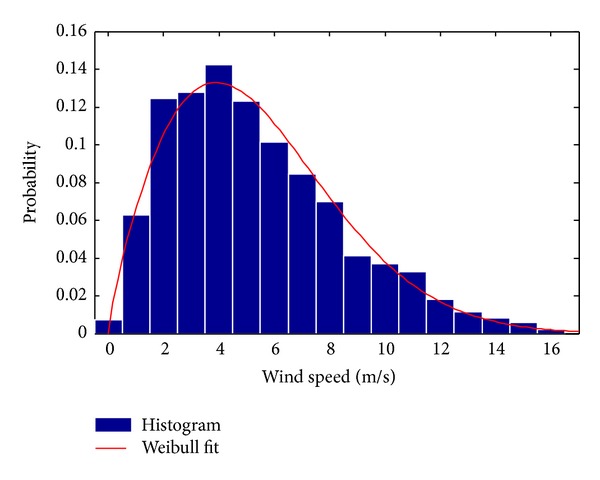
Histogram and Weibull PDF fit of wind speed raw data of Figure 1.

The raw vector can be either transformed into a histogram (discrete PDF) or fitted to a known continuous PDF, typically of Weibull type, as shown in Figure 2. When creating a histogram, the bins are typically chosen to be 1 m·s^−1^ wide to match the resolution of the manufacturer-provided turbine power curve data (explained in Section 3), resulting in the following discrete PDF:

(2) $$\begin{array}{c} f_{\text{HST}}\left(v\right)=f\left(v_{i}\right),\;v_{i}-0.5\leq v<v_{i}+0.5, \end{array}$$

where *f*(*v*_*i*_) is the magnitude of the histogram bin, centered at *v*_*i*_.

As to Weibull fitting, several methods of deriving Weibull parameters from the raw data were compared in [9]. The maximum likelihood estimates of the Weibull distribution parameters are typically employed in dedicated software packages, for example, MATLAB.

Weibull PDF is defined as

(3) $$\begin{array}{c} f_{\text{WBL}}\left(v\right)=\frac{k}{c}{\left(\frac{v}{c}\right)}^{k-1}e^{-{\left(v/c\right)}^{k}} \end{array}$$

with parameters *c* and *k* being related to the site wind speed mean and standard deviation as

(4) $$\begin{array}{c} \mu_{v}=c\Gamma \left(1+\frac{1}{k}\right),\\ \sigma_{v}=c\sqrt{\Gamma \left(1+\frac{2}{k}\right)-\Gamma^{2}\left(1+\frac{1}{k}\right)}, \end{array}$$

respectively, where

(5) $$\begin{array}{c} \Gamma \left(x\right)=\overset{\infty }{\underset{0}{\int }}t^{x-1}e^{-t}dt \end{array}$$

is the complete Gamma function. In case the wind raw data of a site is absent, but the mean and standard deviation of the wind speed are known, Weibull PDF is usually assumed and its parameters are calculated using (4).

A particular (and very common) case of Weibull PDF with *k* = 2 is called Rayleigh PDF and is given by

(6) $$\begin{array}{c} f_{\text{RLH}}\left(v\right)=\frac{2v}{c^{2}}e^{-{\left(v/c\right)}^{2}} \end{array}$$

with scale parameter *c* being related to the site mean wind speed as

(7) $$\begin{array}{c} \mu_{v}=\frac{\sqrt{\pi }}{2}c. \end{array}$$

## 3. Turbine Power Curve

**Figure 3:**
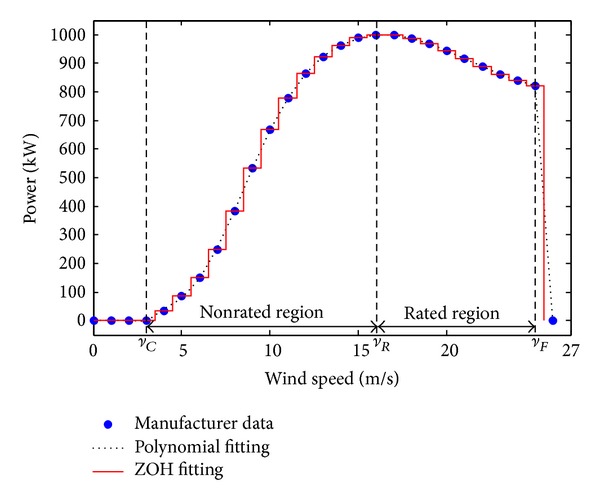
Power curve of NEG Micon 1000/60 fixed speed wind turbine.

The power production of a wind turbine is associated with one of the two nonzero regions of the power curve: the nonrated region for wind speeds between the cut-in speed *v*_*C*_ and the rated speed *v*_*R*_ or the rated region for wind speeds between the rated speed and the furling (or cut-out) speed *v*_*F*_. The turbine power curve is usually supplied by the manufacturer as *N*-point discrete series {*v*_*i*_, *P*(*v*_*i*_)}, *i* = 1,…, *N*, with *v*_*i*_ − *v*_*i*−1_ = 1 for all *i*; that is, the data resolution is 1 m·s^−1^. For example, consider a NEG Micon 1000/60 fixed speed turbine power curve, given in Table 1. The turbine is stall regulated; therefore, the output power in the rated region reduces with the increase of wind speed, as shown in Figure 3.

The power curve data, provided by the manufacturer, may be extrapolated using either Zero Order Hold (ZOH) method [4] or polynomial fitting [3], as shown in Figure 3. In the former case, each data point *P*(*v*_*i*_) is replaced by a 1 m·s^−1^ wide discrete bin, having the same magnitude, thus creating the following staircase approximation of the power curve:

(8) $$\begin{array}{c} P_{\text{ZOH}}\left(v\right)=P\left(v_{i}\right),\;v_{i}-0.5\leq v<v_{i}+0.5, \end{array}$$

for *i* = 1,…, *N*.

In the latter case, the data is divided into nonrated and rated subsets and each subset is fitted to a distinct polynomial, creating the following polynomial approximation of the power curve:

(9) $$\begin{array}{c} P_{\text{POLY}}\left(v\right)=P_{R}\{\begin{array}{cc} 0, & v<v_{C}\\ \overset{n}{\underset{i=0}{\sum }}a_{1i}v^{i}, & v_{C}\leq v\leq v_{R}\\ \overset{n}{\underset{i=0}{\sum }}a_{2i}v^{i}, & v_{R}\leq v\leq v_{F}\\ 0, & v>v_{F}. \end{array} \end{array}$$

## 4. Capacity Factor Calculation

In case the raw wind data of the site at hub height is available, a discrete PDF in the histogram form, described by (2), may be constructed. Hence, combining (2) and (8) as

(10) $$\begin{array}{c} P_{\text{ZOH}}\left(v\right)f_{\text{HST}}\left(v\right)≅P\left(v_{i}\right)f\left(v_{i}\right),\;v_{i}-0.5\leq v<v_{i}+0.5, \end{array}$$

and substituting into (1), the quasiexact solution for capacity factor is given by

(11) $$\begin{array}{c} {\text{CF}}_{\text{QE}}=\frac{1}{P_{R}}\overset{N}{\underset{i=1}{\sum }}P\left(v_{i}\right)f\left(v_{i}\right). \end{array}$$

The approximate solution for capacity factor is obtained by substituting (3) and (9) into (1) as [3]

(12) CFAN=−∑i=0na2ivFie−(vF/c)k−∑i=1nciikΓ(ik)×[a1iγ((vCc)k,ik)−(a1i−a2i)     ×γ((vRc)k,ik)−a2iγ((vFc)k,ik)],

where

(13) $$\begin{array}{c} \gamma \left(y,x\right)=\frac{1}{\Gamma \left(x\right)}\overset{y}{\underset{0}{\int }}t^{x-1}e^{-t}dt \end{array}$$

is the incomplete Gamma function.

As demonstrated in [4], CF against *μ*_*v*_ of any horizontal wind turbine under Rayleigh winds possesses S-shaped behavior with a linear region in the range of average wind speeds. In this linear region, the CF was shown to obey the following relation:

(14) $$\begin{array}{c} \text{CF}=0.087\mu_{v}-\frac{P_{R}}{D^{2}}, \end{array}$$

with *μ*_*v*_ given in m/s, *P*_*R*_, kW, and the rotor blade diameter *D*, m. In the range of capacity factors of 0.15 to 0.45, (14) was shown to be accurate to within 10% for eight turbines of various rated powers and rotor diameters. Nevertheless, this simple CF relationship, while being very handy since it only requires the rated power and rotor diameter for the wind turbine and average site wind speed, is only valid for a limited range of wind speeds. Outside this range, the CF versus average wind speed curve can be no longer approximated by the derived linear relationship. In order to approximate the CF curve for the whole range of average wind speeds, the following nonlinear relationship was proposed in [5]:

(15) CFAP=(1−0.087)·[tanh(0.087μv22π(1+PR/D2)+PR/2D2)−0.0872π(1−PR/D2)μv].

Although nonlinear, (15) requires rated power and rotor diameter for the wind turbine and average site wind speed only, similar to (14). Calculating the CF using (15) is referred to as “approximate” approach in the paper.

## 5. Case Study

Consider a NEG Micon 1000/60 fixed speed stall-controlled wind turbine planned to operate under the wind conditions of Ariel, Israel. The turbine parameters are summarized in Table 2 and its power curve is given in Table 1. Coefficients of fitting the turbine power curve to 6th-order polynomials are shown in Table 3.

**Figure 4:**
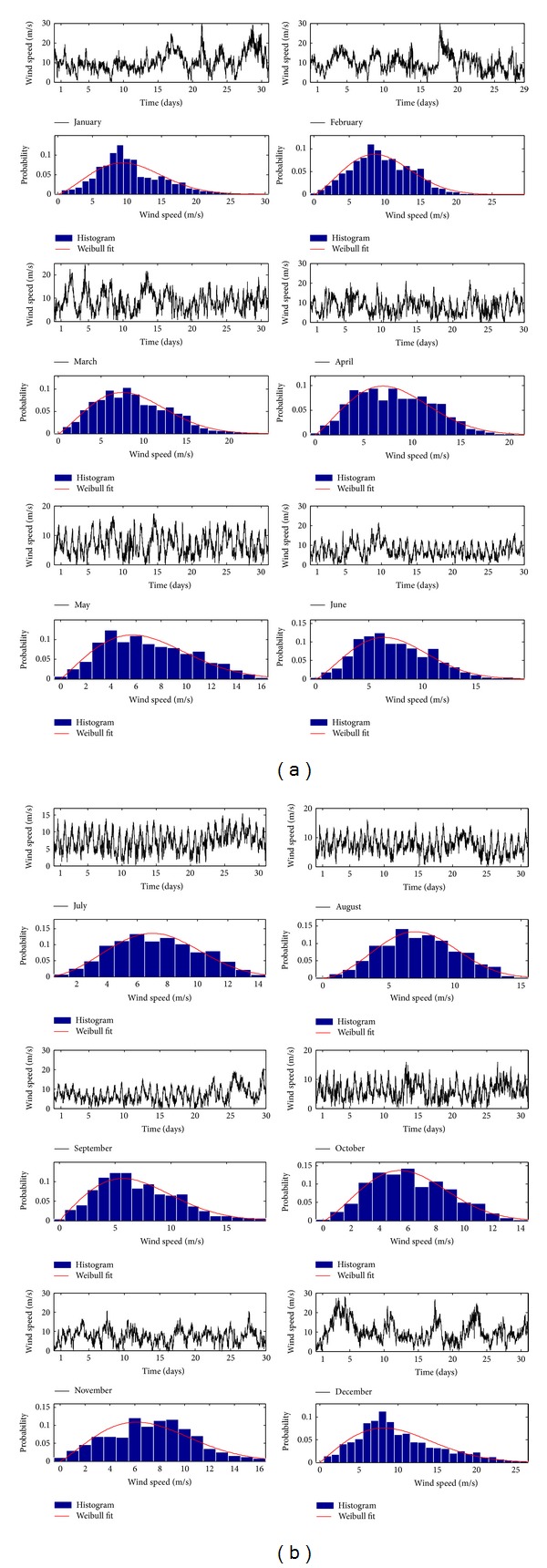
Monthly 2008 wind raw data in Ariel and appropriate PDFs.

The statistical parameters of 10 m height winds are summarized in Table 4. The 2008 monthly wind raw data in Ariel (10 min resolution) at 70 m height is shown in Figure 4 along with the appropriate histograms and fitted Weibull PDFs. The 70 m height wind speed data was extrapolated from the 10 m height data, provided by the Israeli Meteorological Service, using the following relation [4]:

(16) $$\begin{array}{c} \frac{v_{70}}{v_{10}}={\left(\frac{70}{10}\right)}^{0.3}. \end{array}$$

**Figure 5:**
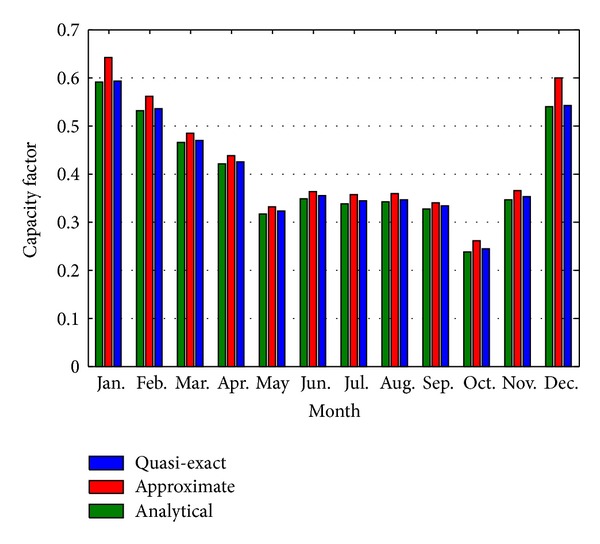
2008 monthly capacity factors.

The resulting “quasiexact,” “analytic,” and “approximate” capacity factors, calculated using (11) and (13), accordingly, are summarized in Table 5 and plotted in Figure 5.

According to Table 5 and Figure 5, the “quasiexact” and “analytical” results are very alike with the largest and smallest errors in October and January, respectively. The “approximate” approach results slightly overestimate the CF since the monthly winds are not exactly Rayleigh with *k* > 2. Two interesting points arise from the case study. First, the quasiexact capacity factor is higher than the analytic throughout the year. Second, the higher the capacity factor is, the lower the difference between the results is; that is, the October capacity factor is the year lowest, while the January capacity factor is the year highest.

## 6. Conclusion

Three approaches to fixed speed wind turbines capacity factor estimation were presented and compared in the paper using a case study. The “quasiexact” approach, which utilizes discrete wind raw and manufacturer-provided turbine power curve data, is only able to numerically calculate the capacity factor. The “analytical” approach, while being an approximation (since it utilizes continuous approximation of wind and power curve data), results in an analytical solution and hence provides a deeper understanding of the factors, affecting the capacity factor and several other merits of wind turbine performance, based on the analytically derived equations. The “approximate” approach, valid in case of Rayleigh winds only, employs a nonlinear approximation of the capacity factor versus average wind speed curve, only requiring rated power and rotor diameter of the turbine. The paper has revealed the close similarity between the results obtained by employing the approaches, enforcing the validity of the analytically derived approximations.

## Figures and Tables

**Table 1:** NEG Micon 1000/60 fixed speed turbine power curve data.

Speed (m·s^−1^)	1	2	3	4	5	6	7	8	9	10	11	12	13
Power (KW)	0	0	0	33	86	150	248	385	535	670	780	864	924

Speed (m·s^−1^)	14	15	16	17	18	19	20	21	22	23	24	25	26
Power (KW)	964	989	1000	998	987	968	944	917	889	863	840	822	0

**Table 2:** NEG Micon 1000/60 fixed speed turbine data.

Cut-in speed, *v*_*C*_ (m·s^−1^)	Rated speed, *v*_*R*_ (m·s^−1^)	Cut-out speed, *v*_*F*_ (m·s^−1^)	Rated power, *P*_*R*_ (KW)	Hub height, *H* (m)
3.5	16	25	1000	70

**Table 3:** 6th-order fitting coefficients of NEG Micon 1000/60 power curve.

*i*	0	1	2	3	4	5	6
a_1*i*_	−2.9	2.3	−0.72	0.11	−9.1 · 10^−3^	0.37 · 10^−3^	−5.8 · 10^−6^
a_2*i*_	12	−3.5	0.45	−0.029	1 · 10^−3^	−0.18 · 10^−4^	1.4 · 10^−7^

**Table 4:** 2008 monthly and yearly 10 m height statistical parameters.

	Jan.	Feb.	Mar.	Apr.	May	Jun.	Jul.	Aug.	Sep.	Oct.	Nov.	Dec.	Year
*c*	6.66	6.04	5.52	5.16	4.40	4.63	4.56	4.59	4.46	3.89	4.62	4.35	5.08
*k*	2.32	2.33	2.19	2.19	2.08	2.26	2.81	2.78	2.02	2.33	2.18	2.03	2.08
*μ*	5.90	5.37	4.88	4.58	3.90	4.11	4.06	4.08	3.96	3.45	4.12	5.62	4.50
*σ*	2.68	2.40	2.36	2.19	1.95	1.92	1.58	1.60	2.04	1.56	1.94	2.91	2.26

**Table 5:** Comparison of 2008 monthly capacity factors.

	Jan.	Feb.	Mar.	Apr.	May	Jun.	Jul.	Aug.	Sep.	Oct.	Nov.	Dec.
CF_QE_	0.5922	0.5341	0.4691	0.4249	0.3225	0.3546	0.343	0.3464	0.3324	0.2437	0.3525	0.5411
CF_AN_	0.5908	0.5302	0.4643	0.4194	0.3165	0.3485	0.3372	0.3405	0.3265	0.2376	0.3465	0.5398
CF_AP_	0.6425	0.5598	0.4834	0.4366	0.3305	0.3633	0.3555	0.3586	0.3399	0.2603	0.3648	0.5988
